# Cellulose Fiber with Enhanced Mechanical Properties: The Role of Co-Solvents in Gel-like NMMO System

**DOI:** 10.3390/gels10090607

**Published:** 2024-09-23

**Authors:** Suhnue Kim, Darae Lee, Hyungsup Kim

**Affiliations:** Department of Materials Science and Engineering, Konkuk University, Seoul 05029, Republic of Korea; jaromekom@konkuk.ac.kr (S.K.); daraelee@konkuk.ac.kr (D.L.)

**Keywords:** cellulose, N-methylmorpholine N-oxide, gel-like solution, co-solvent, conformation, rheology, fiber, mechanical property

## Abstract

Cellulose has garnered attention in the textile industry, but it exhibits limitations in applications that require high strength and modulus. In this study, regenerated cellulose fiber with enhanced mechanical properties was fabricated from a gel-like N-methylmorpholine N-oxide (NMMO)–cellulose solution by modulating the intermolecular interaction and conformation of the cellulose chains. To control the interaction, two types of co-solvents (dimethyl acetamide (DMAc) and dimethyl formamide (DMF)) were added to the cellulose solutions at varying concentrations (10, 20, and 30 wt%). Rheological analysis showed that the co-solvents reduced the solution viscosity by weakening intermolecular interactions. The calculated distance parameter (R_a_) in Hansen space confirmed that the co-solvent disrupted intermolecular hydrogen bonding within cellulose chains. The solutions were spun into fiber via a simple wet spinning process and were characterized by X-ray diffraction (XRD) and universal testing machine (UTM). The addition of co-solvent led to an increased crystallinity index (C.I.) owing to the extended cellulose chains. The modulus of the resulting fiber was increased when the co-solvent concentration was 10 wt%, regardless of the co-solvent type. This study demonstrates the potential for enhancing the mechanical properties of cellulose-based products by modulating the conformation and interaction of cellulose chains through the addition of co-solvent.

## 1. Introduction

Synthetic polymers such as polypropylene, polyethylene terephthalate, and polyurethane dominate the textile industry, accounting for over 60% of the materials used [1]. Due to the growing environmental concerns, studies into alternative materials are ongoing. Cellulose, the most abundant natural polymer, has attracted interest due to its sustainability, biocompatibility, and recyclability. Despite its advantages, the strong inter- and intramolecular hydrogen bonding between cellulose chains prevents it from melting in heat or dissolving in common organic solvents [2]. The traditional way to fabricate cellulose fiber was the viscose process. However, it has environmental disadvantages due to the consumption of carbon disulfide, which is a toxic material [3]. Consequently, studies on eco-friendly solvents for cellulose have been extensively conducted.

As cellulose solvent, many candidates such as LiCl/DMAc, ionic liquid (IL), alkali/urea, deep eutectic solvent (DES), and N-methylmorpholine N-oxide (NMMO) have been explored. However, LiCl/DMAc, alkali/urea, and DES systems suffer from low solubility of cellulose. The low cellulose content leads to insufficient interaction and entanglement between cellulose chains, resulting in poor mechanical properties [4,5,6]. In the case of IL, there are several disadvantages, such as high cost and high processing temperature. Additionally, the high viscosity of the IL itself limits the processing of cellulose solution [7].

Among the various solvents, NMMO is the most commercially successful solvent. The low cost, low toxicity, and good recyclability make NMMO a promising solvent for cellulose, despite requiring high processing temperatures [8]. Zhao et al. reported that depending on the hydration level of NMMO, it can dissolve up to 30 wt% cellulose [9]. Carrillo et al. compared the characteristics of NMMO-based cellulose fiber with viscose-type fiber and showed that NMMO-based fiber has 20% higher crystallinity [10]. However, the strong inter- and intramolecular hydrogen bonding between cellulose chains causes gelation at high concentrations, limiting processing capabilities and preventing higher cellulose concentrations [11]. Consequently, NMMO-based fiber still has insufficient mechanical properties.

The simplest way to control the viscosity of cellulose solutions is to add co-solvents to the solution. Since the co-solvents have very low viscosity compared to other solvents, this can dramatically reduce the viscosity of the solutions. The most commonly used co-solvents for cellulose are dimethyl acetamide (DMAc), dimethyl formamide (DMF), and dimethyl sulfoxide (DMSO) [12,13]. Ahn et al. added DMF and DMAc as co-solvents to IL-based cellulose solution for feasible electrospinning of cellulose and compared the effect of the co-solvents [14]. The addition of co-solvents dramatically enhanced the spinnability of the solution. Additionally, the crystallinity of the fiber was increased when the co-solvents were added. However, the effect of co-solvent on an NMMO–cellulose system remains unexplored.

It is expected that the change in the microstructure of the cellulose fiber is related to the conformation and interaction change between cellulose chains when the co-solvents are added. Rheological analysis is one of the most effective tools to investigate the conformational changes of polymers. It also provides fundamental information about the interaction between the polymer chains [15]. Additionally, the induced shear during measurements can imitate the actual fiber spinning process and is useful for optimizing and controlling the properties of the final products.

In this study, regenerated cellulose fiber with enhanced mechanical properties was fabricated via the introduction of a co-solvent. Due to the better affinity of co-solvents with cellulose compared to NMMO, the addition of co-solvents altered the conformation and interaction of cellulose chains. The conformation and interaction changes in cellulose chains in the solution were investigated by rheological observations and Hansen solubility parameter calculations. The effect of co-solvent on the microstructure and mechanical properties of the regenerated fiber was studied. This study will be helpful as a fundamental study for controlling the mechanical properties of cellulose-based products by introducing co-solvents.

## 2. Results and Discussion

### 2.1. Rheological Behaviors

**Figure 1:**
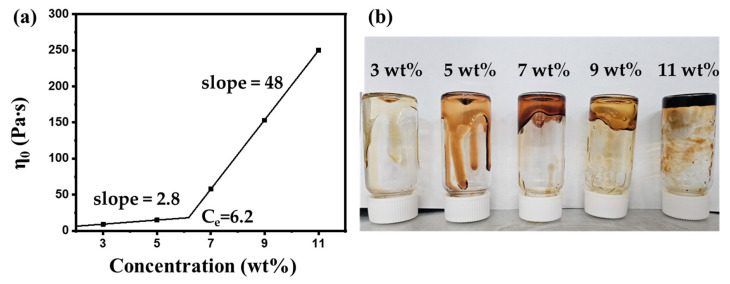
Rheological properties of solutions with different cellulose concentrations. (**a**) Zero-shear viscosity. (**b**) Vial inversion test of each solution.

The rheological behaviors of cellulose solutions with varying cellulose concentrations are shown in Figure 1. The zero-shear viscosity of the solutions is plotted against cellulose concentration in Figure 1a. Typically, zero-shear viscosity is calculated using the Cross model [16]; however, in this study, the viscosity at shear rate 0.01 s^−1^ was used as the zero-shear viscosity due to the absence of a plateau at low shear rates for the solutions with low cellulose concentrations. The zero-shear viscosity of solutions below 5 wt% cellulose concentration was extremely low and sharply increased from 7 wt%. The slope of the viscosity increase was 2.8 in the low concentration range (3 and 5 wt%) and rose to 48 in the high-concentration range (7–11 wt%), with the calculated critical concentration being 6.2 wt%. This suggests gel-like behavior of the high-concentration solutions. As well known in the literature, the zero-shear viscosity of cellulose solution can be divided into four regions: non-contact, entanglement, anisotropic, and gel [17]. However, the long cellulose chain used in this study (DP: 890) led to significant interaction and entanglement even at low concentrations, and the non-contact region did not appear in the concentration range examined. Additionally, due to the long cellulose chains, the liquid crystal phase did not form, resulting in the absence of an anisotropic region. As a result, the solutions below the critical concentration exhibited entanglement behavior, while the solutions above the critical concentration demonstrated a gel-like characteristic. To confirm the gel-like property of the solutions, a vial inversion test of each solution was conducted, with the digital image shown in Figure 1b. Solutions in the low-concentration range flowed immediately, whereas the solutions in the high-concentration range resisted flow. This provides evidence for the gel-like properties of the high-concentration cellulose solutions. Subsequent experiments were conducted with 9 wt% cellulose solutions, which ensures sufficient entanglement and interaction between the cellulose chains while maintaining moderate viscosity for facile spinning.

**Figure 2:**
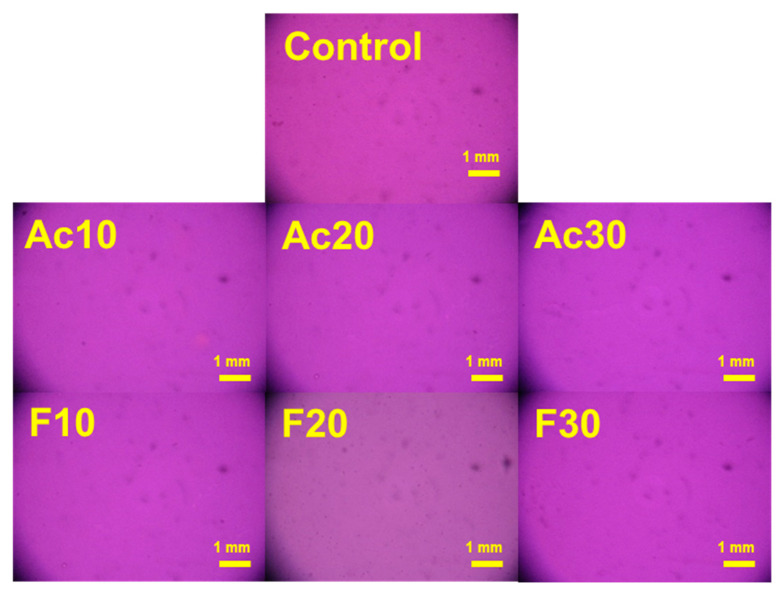
Polarized optical images of cellulose and co-solvent-added solutions. (Control: cellulose sample without co-solvent, Ac: dimethyl acetamide (DMAc), F: dimethyl formamide (DMF), and *x* (10–30): the wt% of the co-solvent).

Figure 2 shows the polarized optical microscopy (POM) images of the solutions. None of the images showed any crystallites or the formation of liquid crystal phases. Given the semi-crystalline nature of cellulose, undissolved cellulose chains would be visible on the image. However, complete dissolution was confirmed, and the addition of co-solvent did not cause any interference. As the undissolved cellulose particles can act as defects in the product due to stress concentration, the thorough dissolution of the cellulose shows the potential of these solutions to be spun into a fiber with sufficient mechanical properties. Although cellulose is well known as a lyotropic biopolymer, the high DP (890) of the cellulose used in this study prevented the formation of liquid crystal phases, as described in Figure 1a [18]. The long polymer chains were unable to achieve the necessary unidirectional orientation for liquid crystal formation.

**Figure 3:**
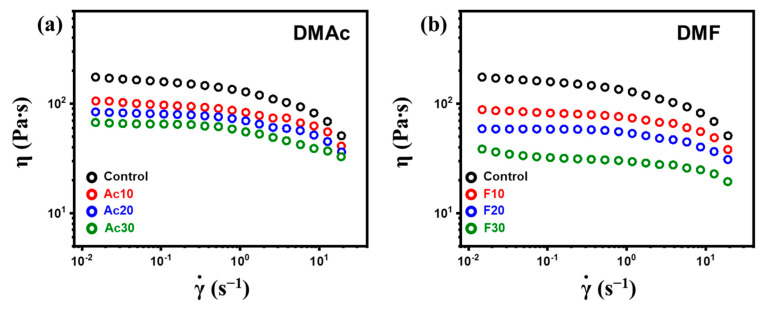
Apparent viscosity of co-solvent-added cellulose solutions. (**a**) Dimethyl acetamide. (DMAc). (**b**) Dimethyl formamide (DMF).

The apparent viscosity of cellulose solutions is shown in Figure 3. All solutions exhibited shear thinning, a typical characteristic of polymer solutions owing to the disentanglement of individual chains [19]. In the low-shear-rate range (ca. ~10^0^ s^−1^), the cellulose chains were entangled with each other and resisted the deformation, resulting in a plateau. However, as the shear rate increased, the cellulose chains were disentangled, and due to the reduced friction, the viscosity of the solution decreased. Upon the addition of co-solvents, the solutions displayed rheological behavior similar to that of the pure cellulose solution, indicating that the co-solvent addition did not significantly affect the physical state of the cellulose chains. The viscosity decreased monotonically as the co-solvent content increased, with DMF-added solutions showing slightly lower viscosity than those with DMAc. This phenomenon will be explained in detail in the next section.

To clarify the effect of co-solvent on the viscosity of the cellulose solutions, the distance parameter (R_a_) from Hansen solubility parameters was calculated. The R_a_ implies the distance between two molecules in Hansen space and is related to the affinity between them [20]. The Hansen solubility parameters of cellulose and the solvents used in this study are listed in Table 1. The R_a_ was determined using Equation (1), where δ_D_, δ_P_, and δ_H_ represent the energy from dispersion forces, dipolar intermolecular forces, and hydrogen bonds, respectively [21].

(1) $${\left(R_{a}\right)}^{2}={4(\delta_{d2}-\delta_{d1})}^{2}+{\left(\delta_{p2}-\delta_{p1}\right)}^{2}+{\left(\delta_{h2}-\delta_{h1}\right)}^{2}$$

As shown in Table 1, the R_a_ value with cellulose decreased in the order of NMMO, DMAc, and DMF, indicating closer interaction between the co-solvents and cellulose than with NMMO. The added co-solvents formed stronger hydrogen bonding with cellulose, reducing the intermolecular hydrogen bonding within the cellulose itself. Additionally, the better affinity of the co-solvents with cellulose compared to NMMO allowed the polymer chains to adopt a more extended conformation in the solutions [22]. As a result, the extension facilitated easier disentanglement of the cellulose chains under shear, due to the slippage between them. The change in the interaction between cellulose chains and the conformation resulted in decreased viscosity. Among the types of co-solvents, DMF showed slightly lower viscosity, reflecting its better affinity with cellulose.

**Figure 4:**
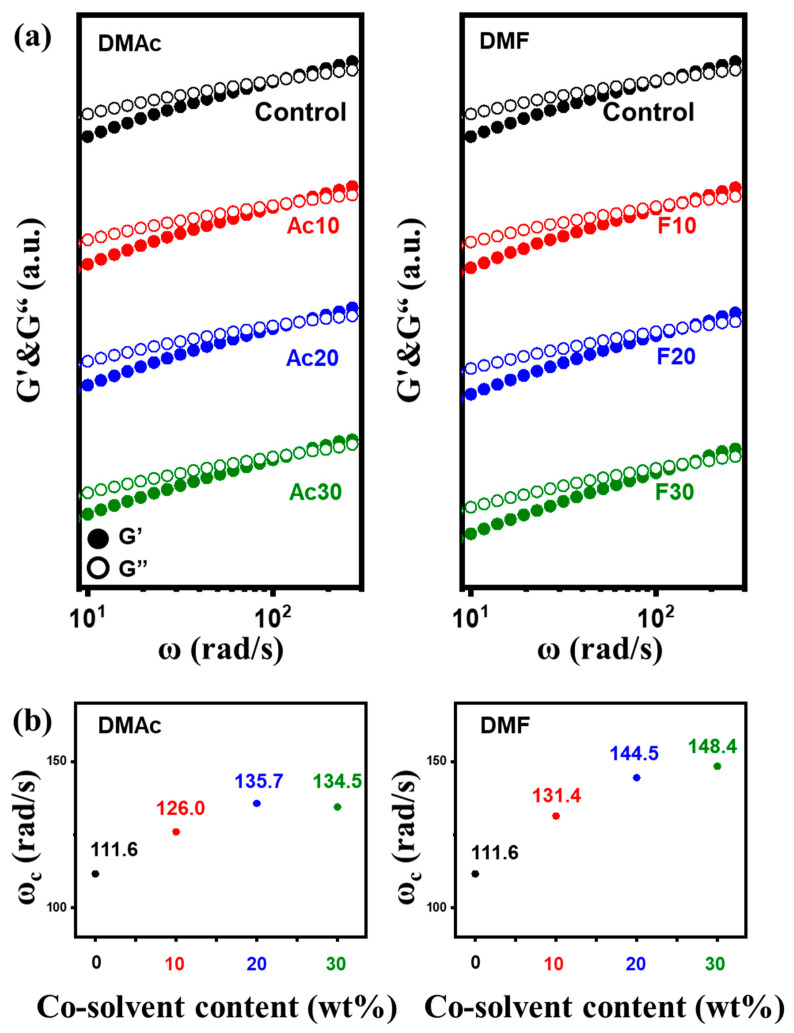
Viscoelastic properties of the solutions. (**a**) Dynamic sweep test results and (**b**) frequency at crossover-point (ω_C_) plotted against the content of the co-solvents.

Dynamic frequency sweep tests were conducted to investigate the viscoelastic behavior of the solutions. In Figure 4a, all solutions show the crossover point of storage (G′) and loss moduli (G″) within the measured frequency range, where G′ and G″ indicate the solid-like and liquid-like properties of the solution, respectively. The presence of a crossover point denotes the entanglement between cellulose chains [23]. This suggests the potential of the solutions for fiber spinning [24]. The frequency at the crossover point (ω_c_) was plotted against the content of the co-solvent in Figure 4b. ω_c_ is one of the most important viscoelastic parameters, and shows the change in the physical behavior of the solutions. The ω_c_ increased as the content of the co-solvent increased. As described above, the co-solvent decreased the intermolecular interaction between cellulose chains and reduced the friction. As a result, co-solvent-added solutions exhibited a more liquid-like behavior compared to pure cellulose solution, particularly in DMF-added solutions.

### 2.2. Characterization of Regenerated Fiber

**Figure 5:**
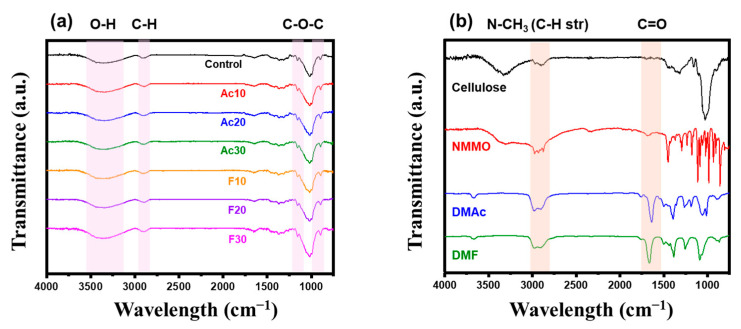
Fourier-transform infrared spectroscopy (FT-IR) graphs of (**a**) regenerated fibers and (**b**) pristine materials.

Figure 5 presents the Fourier-transform infrared spectroscopy (FT-IR) spectra of the regenerated fibers, cellulose pulp, and solvents. In Figure 5a, the regenerated fibers exhibited a broad peak around 3000–3500 cm^−1^ originating from the hydroxyl group and the C-H stretching peak at 2890 cm^−1^. Additionally, C-O-C characteristic peaks were present at 1160 and 897 cm^−1^, indicating that the chemical structure of the regenerated fibers remained unchanged after dissolution and co-solvent addition [25]. In Figure 5b, NMMO shows a characteristic peak of N-CH_3_ at 2875 cm^−1^, while the co-solvents display a carboxyl peak at 1760 cm^−1^ [26,27]. The absence of the NMMO and co-solvent peaks in regenerated fibers (Figure 5a) confirms the effective coagulation with distilled water. As the residual solvents in the fiber can hinder the solidification and act as a plasticizer between the cellulose chains, thorough solvent exchange is essential for achieving good mechanical properties.

**Figure 6:**
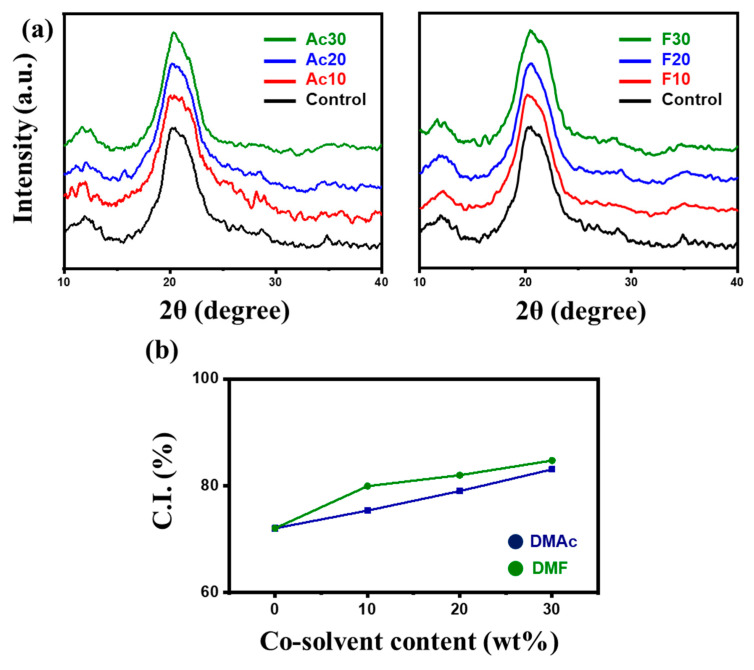
Microstructure of the regenerated fibers. (**a**) X-ray diffraction (XRD) patterns. (**b**) Crystallinity index.

The crystalline structure and crystallinity index (C.I.) of the fibers were investigated using X-ray diffraction (XRD), and the results are shown in Figure 6. All fibers displayed typical cellulose II patterns, with characteristic peaks at 12° and 21°. As well known in the literature, the crystalline structure of cellulose converts from cellulose I to cellulose II after regeneration [28]. The addition of co-solvent did not affect the crystalline structure of the fiber. The C.I., calculated from the XRD patterns using Equation (2), is plotted in Figure 6b [29].

(2) $$C.I.(\%)=\frac{I_{020}-I_{am}}{I_{020}}\times 100$$

Here, I_020_ is the maximum intensity of the diffraction from the (020) plane at 2θ = 21.49°, and I_am_ is the intensity of the amorphous background scatter measured at 2θ = 18°. The C.I. of the pure cellulose fiber was 71.99%, increasing to 83.04 and 84.69% with the addition of 30 wt% DMAc, and DMF, respectively. This increase in crystallinity is attributed to the conformation change of cellulose chains. As mentioned above in Figure 3, the addition of co-solvent resulted in the extension of individual cellulose chains. During the fiber spinning process, the extended chains were more feasibly aligned along the shear direction and resulted in higher C.I.

**Figure 7:**
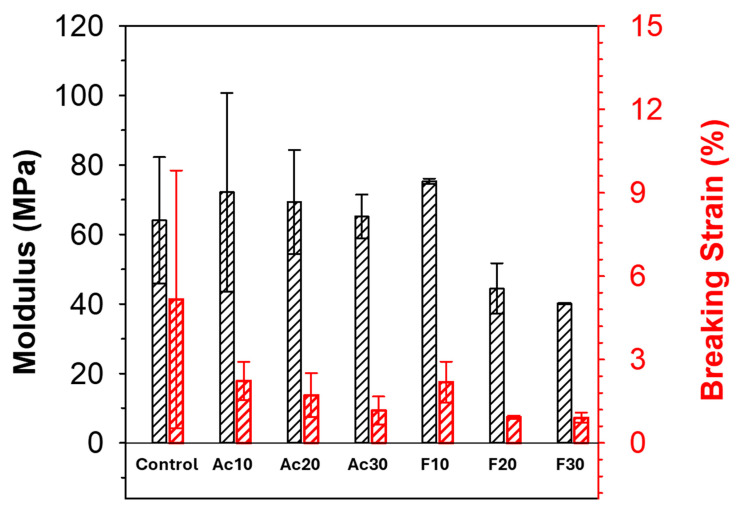
Mechanical properties of the regenerated cellulose fibers.

Figure 7 illustrates the mechanical properties of the regenerated fibers measured using a universal testing machine (UTM). Modulus and strain at breakage are important mechanical parameters related to the crystallinity and entanglement between the polymer chains [30]. The stress–strain curves of the regenerated fibers are shown in Figure S1. The modulus of the regenerated fiber increased when 10 wt% DMF or DMAc was added compared to pure cellulose fiber. This is due to the higher C.I. of the co-solvent-added fiber. As the C.I. of the fiber increased, the fiber was able to resist deformation. However, a further increase in co-solvent content led to a decrease in the modulus. This is likely due to the reduced entanglement between cellulose chains. Although the C.I. of the fiber increased with the content of co-solvent, the entanglement between cellulose chains was reduced due to the interaction between cellulose and co-solvent. The C.I. of the fiber and the entanglement between cellulose chains were inversely related, and 10 wt% co-solvent was the optimum content for the highest modulus. The mechanical properties of the regenerated fibers depended on the type of co-solvent added. Up to 10 wt% DMF showed higher moduli, but DMAc showed higher moduli when the content of co-solvent exceeded 20 wt%. As described in Figure 3, DMF had a more pronounced effect in reducing the entanglement of cellulose chains. As a result, the modulus of DMF-added fiber was lower than DMAc-added fiber. In the case of strain at breakage, it is highly relevant to the C.I., since the fiber becomes more brittle when the C.I. is high. As a result, the strain at breakage of the fibers monotonically decreased as the co-solvent content increased, regardless of the type.

## 3. Conclusions

This study successfully fabricated regenerated cellulose fiber with enhanced mechanical properties. The cellulose solutions above 6.2 wt% concentration showed gel-like behavior due to severe hydrogen bonding between cellulose chains. The co-solvents (DMF and DMAc) were introduced to the 9 wt% cellulose solution in order to reduce the viscosity. POM images confirmed the complete dissolution of cellulose across all solutions. The apparent viscosity of the solution at a 0.01 s^−1^ shear rate decreased significantly from 174 Pa·s to 67 and 38 Pa·s with the addition of 30 wt% DMAc and DMF, respectively. The viscoelastic properties of the co-solvent-added solutions showed a shift towards more liquid-like behavior, as indicated by an increase in ω_c_ values from 111.6 rad/s to 134.5 and 148.4 rad/s for the DMAc- and DMF 30 wt%-added solutions. R_a_ calculation revealed that the co-solvents have a better affinity with cellulose compared to NMMO. Consequently, the hydrogen bonding between cellulose and co-solvents increased and the friction between cellulose chains was reduced. Additionally, slippage between cellulose chains was facilitated due to the extended polymers, resulting in more liquid-like behavior.

The solutions were successfully fabricated into regenerated fibers via a simple spinning process. FT-IR analysis indicated that the chemical structure of cellulose remained unchanged after co-solvent addition. The regenerated fibers showed typical cellulose II peaks, and the C.I. of the fibers proportionally increased with co-solvent content from 71.99% to 83.04 and 84.69% for DMAc and DMF, respectively, owing to the extended cellulose chains. However, the mechanical properties of the fibers did not uniformly improve with higher co-solvent concentrations. Although the C.I. increased, excessive co-solvent reduced cellulose chain entanglement, resulting in decreased moduli. Optimal mechanical properties were achieved at 10 wt% co-solvent, with the highest modulus of 75.31 MPa observed in the fiber containing 10 wt% DMF. This study provides fundamental insights into controlling the rheological behaviors and mechanical properties of cellulose products via co-solvent addition.

## 4. Materials and Methods

### 4.1. Materials

Cellulose pulp (DP: 890) was kindly provided by Seowontech Co. (Daegu, Republic of Korea) and was stored in a 70 °C oven to prevent moisture absorption. N-methylmorpholine N-oxide (NMMO, 50 wt% in H_2_O), propyl gallate (PG, purity ≥ 98%), dimethyl acetamide (DMAc, purity ≥ 99%), and dimethyl formamide (DMF, purity ≥ 99%) were purchased from Sigma Aldrich Co. (St. Louis, MO, USA). The hydration level of NMMO was reduced to 1 via a simple evaporation process at 80 °C for approximately 2 h before use to enhance the dissolution capability of NMMO.

### 4.2. Cellulose Solution and Regenerated Fiber Fabrication

Cellulose solutions with varying concentrations were prepared by dissolving the required amount of cellulose in monohydrate NMMO at 110 °C for 2 h. To prevent cellulose depolymerization, 1 wt% PG was added relative to cellulose weight. In the case of co-solvent-added solutions, 10, 20, and 30 wt% DMAc or DMF was added after 1 h of cellulose dissolution. The total solvent weight including the co-solvent was fixed at 20 g. The sample codes and concentrations for each solution are listed in Table 2.

The prepared solutions were extruded into fibers using a syringe pump (NE-8000, New Era Pump Systems, Inc., Farmingdale, NY, USA) with a 20 G needle (O.D.: 0.908 mm, I.D.: 0.603 mm) at a 1 mL/min rate. Distilled water (D.W.) was used as the coagulation bath, and the fibers were immersed in D.W. for 12 h to completely remove any remaining solvents.

### 4.3. Characterization

Polarized optical microscopy (POM, BX-41, Olympus, Tokyo, Japan) was used to confirm the dissolution of cellulose in NMMO and co-solvent-added solvents. The POM images of the solutions were taken at room temperature (20~25 °C) using a digital camera with a 530 nm sensitive tint plate (U-TP530, Olympus) as a plate compensator to introduce a magenta background in the micrographs. The rheological behaviors of the cellulose solutions were measured using a stress-controlled rheometer (RS-1, Thermo Fisher Scientific, Waltham, MA, USA) at 110 °C with a 35 mm parallel plate. To prevent moisture absorption and solidification of the solution, the open side of the solution sandwiched between the plates was covered with a thin layer of silicone oil (Shin-Etsu Chemical Co., Tokyo, Japan). The steady shear flow test was conducted in a shear rate range of 10^−2^–10^2^ s^−1^. The dynamic frequency sweep was measured in the frequency range of 10^−1^–10^3^ rad/s in the viscoelastic linear region. The HSPiP program (fourth edition 4.1.07, Steven Abbott TCNF Ltd, Ipswich, UK) was used to calculate the Hansen solubility parameters of cellulose and solvents.

The chemical structure of the regenerated fibers was analyzed using FT-IR (FT/IR-4100, attenuated total reflectance mode, Jasco, Tokyo, Japan). The microstructure of the fibers was investigated using XRD (SmartLab, Rigaku, Tokyo, Japan) in the 2θ range of 5–40° with Cu Kα radiation. C.I. based on the Segal method was calculated from the height ratio between the intensity of the crystalline peak (I_020_-I_AM_) and total intensity (I_020_) after subtraction of the background signal. The tensile properties of the films were tested using a UTM (UTM5900, Instron, Norwood, MA, USA) at a test rate of 5 mm/min. Each test was repeated three times.

## Figures and Tables

**Table 1:** Hansen solubility parameters and the calculated distance parameter (R_a_) between cellulose and solvents.

	δ_D_	δ_P_	δ_H_	R_a_
Cellulose	15.6	17.2	20.4	-
NMMO	8.6	8.6	15.4	18.9
DMAc	16.8	11.5	10.2	12.3
DMF	17.4	13.7	11.3	10.4

**Table 2:** Composition of the solvents.

Sample Codes	NMMO Concentration (wt%)	DMAc Concentration (wt%)	DMF Concentration (wt%)	Total Solvent (g)
Control	100	-	-	20
Ac10	90	10	-	20
Ac20	80	20	-	20
Ac30	70	30	-	20
F10	90	-	10	20
F20	80	-	20	20
F30	70	-	30	20

## Data Availability

The data presented in this study are available on request from the corresponding author due to the policy of the funding.

## Supplementary Materials

The following supporting information can be downloaded at https://www.mdpi.com/article/10.3390/gels10090607/s1. Figure S1: Stress-strain curves of the regenerated fibers.

## References

[1] Periyasamy A.P. (2023). Environmentally Friendly Approach to the Reduction of Microplastics during Domestic Washing: Prospects for Machine Vision in Microplastics Reduction. Toxics.

[2] Schroeter J., Felix F. (2005). Melting cellulose. Cellulose.

[3] Chen J., Guan Y., Wang K., Zhang X., Xu F., Sun R. (2015). Combined effects of raw materials and solvent systems on the preparation and properties of regenerated cellulose fibers. Carbohydr. Polym..

[4] Hong Y.-K., Chung K.-H., Lee W.-S. (1998). Structure of regenerated cellulose fibers from DMAc/LiCl solution. Text. Res. J..

[5] Klar V., Orelma H., Rautkoski H., Kuosmanen P., Harlin A. (2018). Spinning Approach for Cellulose Fiber Yarn Using a Deep Eutectic Solvent and an Inclined Channel. ACS Omega.

[6] Cai J., Zhang L., Zhou J., Li H., Chen H., Jin H. (2004). Novel fibers prepared from cellulose in NaOH/urea aqueous solution. Macromol. Rapid Commun..

[7] Wang H., Gurau G., Rogers R.D. (2012). Ionic liquid processing of cellulose. Chem. Soc. Rev..

[8] Rosenau T., Potthast A., Sixta H., Kosma P. (2001). The chemistry of side reactions and byproduct formation in the system NMMO/cellulose (Lyocell process). Prog. Polym. Sci..

[9] Zhao H., Kwak J., Wang Y., Franz J., White J., Holladay J. (2007). Interactions between cellulose and N-methylmorpholine-N-oxide. Carbohydr. Polym..

[10] Carrillo F., Colom X., Suñol J.J., Saurina J. (2004). Structural FTIR analysis and thermal characterisation of lyocell and viscose-type fibres. Eur. Polym. J..

[11] Braverman L., Romanov V., Lunina O., Belasheva T., Finger G. (1990). Rheological properties of concentrated cellulose solutions in N-methylmorpholine-N-oxide. Fibre Chem..

[12] Han S.-Y., Park C.-W., Febrianto F., Kim N.-H., Lee S.-H. (2020). Pretreatment with [EMIM] Ac/DMAc co-solvent to improve enzymatic saccharification of pussy willow (*Salix gracilistyla* Miq.). BioResources.

[13] Byrne N., Leblais A., Fox B. (2014). Preparation of polyacrylonitrile–natural polymer composite precursors for carbon fiber using ionic liquid co solvent solutions. J. Mater. Chem. A.

[14] Ahn Y., Hu D.H., Hong J.H., Lee S.H., Kim H.J., Kim H. (2012). Effect of co-solvent on the spinnability and properties of electrospun cellulose nanofiber. Carbohydr. Polym..

[15] Alves L., Magalhaes S., Pedrosa J.F.S., Ferreira P.J.T., Gamelas J.A.F., Rasteiro M.G. (2024). Rheology of Suspensions of TEMPO-Oxidised and Cationic Cellulose Nanofibrils-The Effect of Chemical Pre-Treatment. Gels.

[16] Liao J., Pham K.A., Breedveld V. (2020). Rheological characterization and modeling of cellulose nanocrystal and TEMPO-oxidized cellulose nanofibril suspensions. Cellulose.

[17] Kim T., Song Y., Ahn J., Kim M., Ko E., Kim H. (2021). Rheological interpretation of intermediate physical state of gel and liquid crystalline phases in cellulose solution and their synergetic effects on the mechanical property. Cellulose.

[18] Gilbert R.-D., Patton P. (1983). Liquid crystal formation in cellulose and cellulose derivatives. Prog. Polym. Sci..

[19] Chaffey C. (1977). Mechanisms and equations for shear thinning and thickening in dispersions. Colloid Polym. Sci..

[20] Saiz C.A., Darvishmanesh S., Buekenhoudt A., Van der Bruggen B. (2018). Shortcut applications of the Hansen solubility parameter for organic solvent nanofiltration. J. Membr. Sci..

[21] Hussain A., Altamimi M.A., Ramzan M., Mirza M.A., Khuroo T. (2023). GastroPlus-and HSPiP-oriented predictive parameters as the basis of valproic acid-loaded mucoadhesive cationic nanoemulsion gel for improved nose-to-brain delivery to control convulsion in humans. Gels.

[22] Chremos A., Douglas J.F. (2018). The influence of polymer and ion solvation on the conformational properties of flexible polyelectrolytes. Gels.

[23] Lu M., Liao J., Gulgunje P.V., Chang H., Arias-Monje P.J., Ramachandran J., Breedveld V., Kumar S. (2021). Rheological behavior and fiber spinning of polyacrylonitrile (PAN)/Carbon nanotube (CNT) dispersions at high CNT loading. Polymer.

[24] Zhu Y., Wu C., Zhang Y., Zhao J. (2015). Study on the chain entanglement of polyvinyl alcohol fiber during the dry-jet wet spinning process. Fibers Polym..

[25] Morán J.I., Alvarez V.A., Cyras V.P., Vázquez A. (2008). Extraction of cellulose and preparation of nanocellulose from sisal fibers. Cellulose.

[26] Smith B. (2019). Organic nitrogen compounds III: Secondary and tertiary amines. Spectroscopy.

[27] Ren Y.-K., Liu S.-D., Duan B., Xu Y.-F., Li Z.-Q., Huang Y., Hu L.-H., Zhu J., Dai S.-Y. (2017). Controllable intermediates by molecular self-assembly for optimizing the fabrication of large-grain perovskite films via one-step spin-coating. J. Alloys Compd..

[28] Cao X., Peng X., Sun S., Zhong L., Wang S., Lu F., Sun R. (2014). Impact of regeneration process on the crystalline structure and enzymatic hydrolysis of cellulose obtained from ionic liquid. Carbohydr. Polym..

[29] French A.D., Santiago Cintrón M. (2013). Cellulose polymorphy, crystallite size, and the Segal Crystallinity Index. Cellulose.

[30] He T. (1987). Polymer strength and chain conformation. Die Makromol. Chem. Macromol. Chem. Phys..

