# Supplementary material for: Cellulose Fiber with Enhanced Mechanical Properties: The Role of Co-Solvents in Gel-like NMMO System
Source: Gels. 2024 Sep 23;10(9):607. doi: 10.3390/gels10090607 (PMC11430876; doi:10.3390/gels10090607)
Supplement: Supplementary file 1 [file gels-10-00607-s001.zip › gels-3207940-supplementary.pdf]

gels

MDPI

Article

Cellulose Fiber with Enhanced Mechanical Properties: The Role of Co-Solvents in Gel-like NMMO System

Suhnue Kim [1], Darae Lee [1] and Hyungsup Kim [1,]*

[1] Department of Materials Science and Engineering, Konkuk University, Seoul 05029, Republic of Korea; jaromekom@konkuk.ac.kr (S.K.); daraelee@konkuk.ac.kr (D.L.)
* Correspondence: iconclast@konkuk.ac.kr

This PDF file includes:

Figure S1

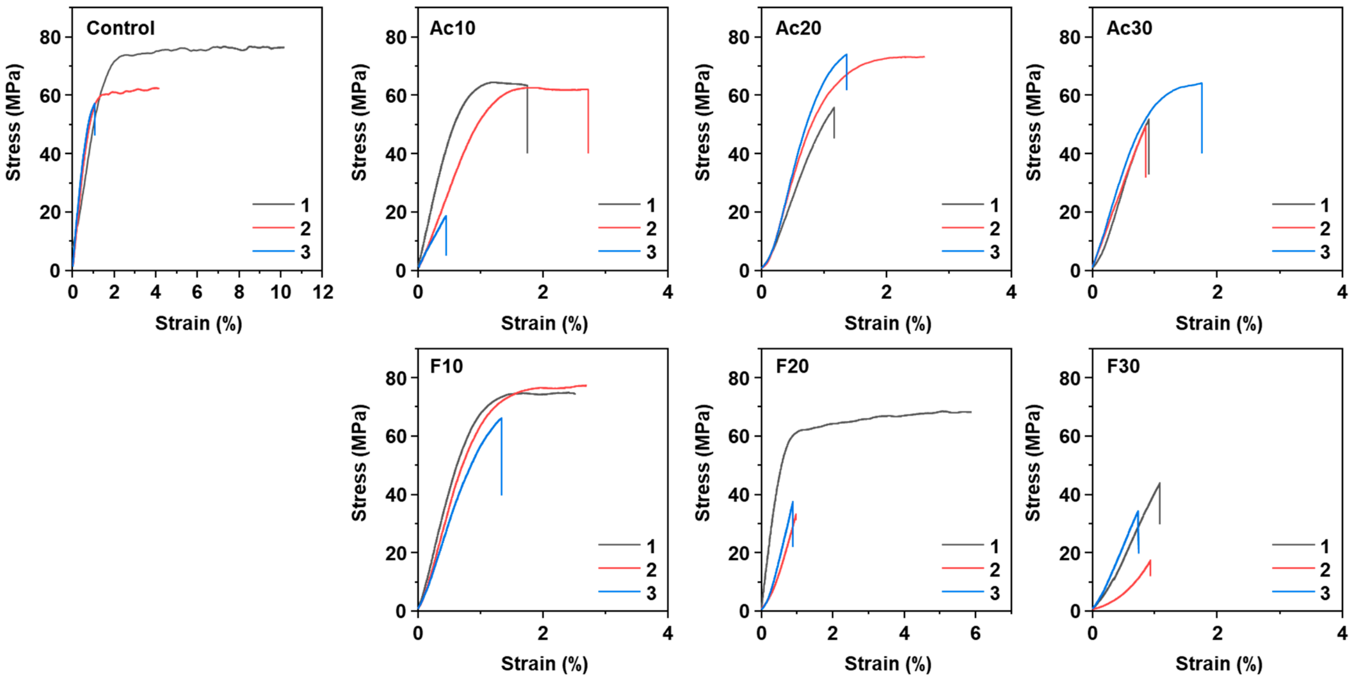

Figure S1. Stress-strain curves of the regenerated fibers.
